# Retraction: MiR-145 Regulates Epithelial to Mesenchymal Transition of Breast Cancer Cells by Targeting Oct4

**DOI:** 10.1371/annotation/aaef10de-ee36-416f-93eb-959dfa3dc92e

**Published:** 2013-05-20

**Authors:** Jiajia Hu, Hua Guo, Hongyan Li, Yan Liu, Jingjing Liu, Liwei Chen, Jin Zhang, Ning Zhang

The editors and the authors retract this publication following the authors’ request. 

The following concerns were raised to the editors’ attention:

1. The legend for Figure 3 does not match the content of the figure.

2. Figure 2C is very similar to Figure 3A.

3. Figure 3C is very similar to the middle panel for Figure 5B.

4. In Figure 4E, the bands for ZEB1 look very similar to those for Snail WB in ZR-75-30 cells.

In response to the editors’ request for the raw data and a clarification in relation to the concerns raised, the authors indicated that they did not have a full set of raw data for the figures in the article. As a result, the authors requested the retraction of the publication.

The editors referred this matter to Tianjin Medical University to request an investigation into the concerns identified about the article and the lack of appropriate records for the data reported. The representatives of Tianjin Medical University have completed their investigation and they support the authors’ request for the retraction of the article.

